# Non-parental Adults and Sexual Health Behaviors Among Young Minority Men: A Qualitative Examination

**DOI:** 10.3389/fpsyg.2021.598120

**Published:** 2021-12-02

**Authors:** Emma M. Sterrett-Hong, Joseph DeBow, Erica Caton, Matthew Harris, Russell Brewer, Erin Roberts, Madeline Marchal, Marjorie Tauzer, Emily A. Arnold

**Affiliations:** ^1^Kent School of Social Work, University of Louisville, Louisville, KY, United States; ^2^Center for the Advancement of Teaching, Florida International University, Miami, FL, United States; ^3^Department of Medicine, University of Chicago, Chicago, IL, United States; ^4^Marriage and Family Therapy Program, East Carolina University, Greenville, NC, United States; ^5^Center for AIDS Prevention Studies, University of California, San-Francisco, San Francisco, CA, United States

**Keywords:** HIV, youth, ethnicity, social support, LGBTQ, non-parental adults

## Abstract

Young Black and Latino sexual minority men (YBLSM) exhibit disproportionately high rates of negative sexual health outcomes, including HIV and other sexually transmitted infections, compared to other groups, partly due to relatively higher rates of exposure to a host of socio-structural risk factors (e.g., unstable housing and under-employment). However, an under-studied interpersonal resource exists for many YBLSM, non-parental adults (NPAs, i.e., adults who act as role models and provide social support), who may be able to influence contextual (e.g., unemployment) and individual (e.g., reduced health expectations) factors underlying sexual health disparities.

**Aims:** This study sought to examine the role of NPAs in factors that affect sexual health behaviors and in supporting those health behaviors directly, among YBLSM living in a mid-sized city in the southern United States. A total of *n*=20 participants, *n*=10 YBLSM (ages 16 to 22), and *n*=10 NPAs (ages 26 to 52) were interviewed using semi-structured guides to examine NPA involvement in the lives of YBLSM from both sides of the relationship. The research team used a framework analysis approach to iteratively identify and define meaningful codes and sub-codes. Both YBLSM and NPAs described NPAs helping YBLSM through role modeling and social support in a variety of areas found to affect sexual health behaviors, such as housing instability and psychological distress, as well as in specific behaviors, such as condom use and HIV medication adherence. Given the multiple socio-structural obstacles facing YBLSM and their multifaceted relationships with NPAs, NPAs may be a promising resource to help address these impediments to health. Partnering more intentionally with NPAs is a potentially promising strategy to help reduce HIV-related disparities affecting YBLSM that is worthy of additional empirical attention.

## Introduction

Young Black and Latino sexual minority men (YBLSM) face disproportionate rates of negative sexual health indicators. In 2019, among sexual minority men ages 13 to 24, YBLSM made up 81% of new HIV diagnoses (Centers for Disease Control and Prevention, 2019). It is estimated that, during their lifetime, 41% of Black and 22% of Latino sexual minority males compared to 9% of white sexual minority males will be diagnosed as HIV-positive (Hess et al., 2017). In addition, sexual minority males, in general, are disproportionately impacted by other sexually transmitted diseases (STDs), including accounting for nearly half of syphilis cases in the United States in 2019 (Centers for Disease Control and Prevention, 2021). In addition, the rate of STDs among all Black Americans is five to eight times the rate of White Americans, and the rate among Latinx Americans is one to two times higher than that of White Americans (Centers for Disease Control and Prevention, 2021).

A number of socio-structural and individual risk factors underlie these sexual health disparities. As many as 29% of lesbian, gay, bisexual, and transgender (LGBT) youth are kicked out or run away from home due to family rejection (The Trevor Project, 2020). Some evidence suggests ethnic minority LGBT youth experience disruptions in relationships with parents at even higher rates than Caucasian LGBT youth. A recent study found that both ethnic minority parents and their sexual minority adolescent children reported higher rates of parental rejection in response to sexual minority orientation disclosure than Caucasian parents and their sexual minority adolescents (Richter et al., 2017). The majority of LGBT youth also have faced sexual orientation-related victimization in high school from peers, both blatantly and in the forms of more subtle microaggressions, and ethnic minority LGBT youth face both sexual orientation and race-based discrimination (Jamil et al., 2009; Baricevic and Kashubeck-West, 2019; Truong et al., 2020).

According to Social Action Theory (SAT), a socio-ecological model of health disparities, background factors, such as societal influences and marginalized identities, affect individual self-change factors, such as motivation, that serve as the primary mechanisms for influencing sexual health behaviors (Lightfoot et al., 2007; Hussen et al., 2015). SAT has been utilized to understand a variety of sexual health behaviors, including condom use and repeated HIV testing (McNairy and El-Sadr, 2014; Holloway et al., 2017).

A second body of literature has documented key resilience factors in sexual minority communities, specifically, the existence of individuals who are part of a “chosen” or “gay” families, including individuals who may serve as “gay mothers” or “gay fathers” regardless of gender identity (e.g., a gay mother who is male-identified; Weston, 1997; Kuvalanka, 2012). Empirical work with LGBT adolescents also highlights the benefits of adults other than caregivers who provide support and acceptance (Bird et al., 2012; Mulcahy et al., 2016; Sterrett-Hong et al., 2020). The importance and value of adults outside of parents or caregivers in supporting youth development in both Black and Latinx cultures also has been elucidated (Comeau, 2012; Taylor et al., 2013; Carey, 2016). In the broader adolescent and young adult development literature, non-parental adults (NPAs) or adults outside of caregivers whom a young person identifies as someone they look up to and who provides social support have been identified as a health promoting factor (Duke et al., 2017; Yu et al., 2019). Mentoring theory suggests that NPAs can exert influence on youth and young adults through a combination of acting as a role model, or embodying characteristics or accomplishments they would like to achieve, along with providing various forms of social support, including emotional support (i.e., nurturance, expressions of caring), tangible support (i.e., provision of concrete goods), esteem support (i.e., instilling confidence in positive qualities), informational support (i.e., advice and guidance), and instrumental support (i.e., engaging in helpful services and actions), that help young people develop emotionally, cognitively, and socially (Rhodes et al., 2006; Sterrett et al., 2011; Hagler, 2018).

While it is becoming clear that a combination of socio-structural and individual-level factors underlies the disproportionate rates of HIV affecting YBLSM, examinations of naturally occurring resilience factors that may help mitigate HIV vulnerability are limited. Guided by both mentoring theory and social action theory, this paper seeks to answer the following two research questions: (1) In what ways do NPAs affect structural and individual drivers of HIV incidence and morbidity among YBLSM? and (2) In what ways do NPAs affect HIV-related health behaviors among YBLSM?

## Materials and Methods

### Ethics

The University of Louisville Institutional Review Board approved all study procedures prior to the initiation of data collection. Participants age 18 and older provided their written informed consent to participate in this study. To protect the privacy of participants age 16 and 17 with regard to their sexual orientation and reduce the risk for any potential parental abuse that might occur as a result of disclosure of a sexual minority status, participants age 16 and 17 whose parents were unaware of their sexual orientation provided their written assent, and an adult at school or a community agency acted as a youth advocate on their behalf. The youth advocate was present, while the study was explained to the youth and remained in a separate room on the premises during the interview. The participants were informed they could contact the youth advocate if they had questions or concerns during the interview.

### Study Overview

This study sought to examine NPA roles and influence on sexual health behaviors among YBLSM. Data for this study are drawn from two sources. Individual semi-structured interviews were conducted with YBLSM who reported having at least one NPA in their life. To better understand the relationships from the perspective of NPAs, approximately a year later, a second set of interviews were conducted, these with NPAs who were not necessarily NPAs of the YBLSM interviewed, but who served in that role for other YBLSM. All participants resided in a medium-sized city in the southern United States. At the time of the study, the majority of the high schools in the city, including the one where some participants for the study were recruited, did not have formal support groups or gay-straight alliances for LGBTQ+ students. Only one of the four-year universities in the city had an LGBTQ+ center for students.

### Recruitment

The research team recruited participants from a university LGBTQ Center, an LGBTQ community organization, local LGBTQ bars and clubs, the local Pride parade, a high school, and a university-based HIV clinic. The research team also utilized peer recruitment by asking each participant (both YBLSM and NPAs) if they knew of anyone personally who may qualify for the study.

### Inclusion Criteria

Inclusion criteria for YBLSM participants included people who were: between the ages of 16 and 22; and identified as Black, Latino, or multi-ethnic including Black and/or Latino; male; and as gay, bisexual, or otherwise identified sexual minority; and had at least one NPA in their lives, defined as someone at least five years older than them who provided them with a support and was not a parent or romantic or sexual partner. Inclusion criteria for NPAs included: adults over the age of 18, reported having a supportive relationship with a YBLSM who is at least five years younger and not a child or romantic/sexual partner. The eligibility of both YBLSM and NPAs was confirmed by asking whether they met the respective inclusion criteria prior to obtaining consent and assent.

### Participants

A total of *n*=10 YBLSM between the ages of 16 and 22 participated. Seven of the participants were Black, two Latino, and one bi-racial. Five YBLSM lived with their biological parents, one lived with a grandparent, and four lived in university dorms or apartments. In addition, a total of *n*=10 NPAs participated. NPAs ranged in age from 26 to 52, with eight NPA participants identifying as Black and two as White. The way in which they met the majority of YBLSM to whom they provided support included in their roles as gay/chosen family members (*n*=4), university staff members (*n*=2), older friend (*n*=1), biological aunt (*n*=1), staff member at a non-profit community organization (*n*=1), and pastor (*n*=1).

### Data Collection

After obtaining informed consent, the first author, an African-American/Latina mostly straight cisgender woman, used semi-structured interview guides to interview participants from 2013 to 2016. Interviews were conducted at various settings, including schools, community settings, and participant homes. The interview guides for YBLSM first asked them to list the initials of all the people in their life who met the definition of an NPA (i.e., at least five years older and not a parent or romantic/sexual partner). Next, YBLSM were asked questions about the overall role of NPAs in their lives, such as “How have NPAs been helpful to you?” and “Do you think NPAs have had any influence on your decision to practice safer sex? If so, how?” The focus of the first phase of data collection with YBLSM was on the influence of NPAs on overall health and well-being, and thus, they were not asked to report on their HIV status. During interviews with NPAs, NPAs were first asked to think about h YBLSM to whom they had provided support. They were then asked questions about the relationship such as, “In what ways do you provide mentorship or support to them?” and “What sorts of things do you do together with the young man?” Since NPAs were asked to describe their relationships with multiple YBLSM, and as most NPA participants described providing specific HIV-related support to YBLSM who were HIV-positive, as well as a variety of types of supports to YBLSM who were HIV-negative, a question related to supporting YBLSM who were HIV-positive was added to the NPA interview guide in later interviews. Interviews lasted between 30 and 60min and were audio-recorded. YBLSM were compensated $25, and NPAs were compensated $30 for their time. All interviews were professionally transcribed verbatim, and transcripts were loaded into Dedoose, a qualitative data analysis platform.

### Method of Analysis

A framework analysis approach, a structured method of qualitative analysis to categorize and organize data (Gale et al., 2013), was used. Framework analysis is appropriate when the research questions are fairly focused and the data is not considerably heterogeneous (Gale et al., 2013). The coding team, consisting of four graduate students, two research staff, and the first author, began analyzing the transcript data through line-by-line reading of the interviews. *A priori* concepts in mentoring theory (e.g., social support types and role modeling), as well as factors that influence sexual health behaviors highlighted in Social Action Theory (e.g., contextual influences and individual influences), were used as a guide in identifying meaningful words and phrases. Team members wrote memos during open coding, documenting thoughts and reactions to the data. The open coding led to the identification of two major themes (e.g., stigma/victimization/rejection), and a list of initial sub-themes, or codes (e.g., inspiration/role model and social support). Next, we iteratively defined and refined the codes as we applied them to different excerpts in the transcript and also identified sub-code (e.g., concrete support) instances of a concept. To establish inter-coder agreement, the first and second author coded a subset of transcripts independently and achieved agreement scores ranging from 84 to 100% on each code. Next, the first and second author independently coded the remainder of the transcripts and also continued recording memos of the variety of types of data that fit under each code (e.g., concrete support related to school, housing, and HIV) and connections between codes. Thematic saturation was reached on the major themes. The research team conducted member checking by presenting the codes and sub-codes, along with their definitions, to four YBLSM and four NPAs and soliciting their feedback on the extent to which they accurately reflect their experiences.

## Results

YBLSM varied in the number of NPAs they reported being in their lives, although most described one to three NPAs who were most influential. The ways in which YBLSM met NPAs also varied. People described by YBLSM as NPAs were parents of friends, school personnel (e.g., teachers and counselors), biological family members (e.g., grandmothers), and chosen family members (e.g., gay/house mothers). NPA participants also varied in the number of YBLSM they had supported. The biological family member reported supporting just one YBLSM, her nephew, whereas most chosen family member NPA participants described supporting at least five or six YBLSM. Frequency of contact varied over the course of relationships and across participants, although most YBLSM and NPA participants described interacting at least weekly at one point in YBLSM-NPA relationships described as being the closest. Four YBLSM participants described at least one relationship with an NPA who enrolled in the second phase of the study.

We identified two major or parent themes in interviews with YBLSM and NPAs. The first parent theme, stigma/discrimination/rejection, captures oppression experienced by YBLSM. A second theme, NPA impact on sexual health, included NPA influence on contextual (e.g., housing) and individual (e.g., mental health) factors that affect sexual health behaviors, as well as influencing sexual health behaviors directly.

### Stigma/Discrimination/Rejection

Consistent with other studies with ethnic and sexual minority males (Puckett et al., 2015; Quinn et al., 2019), several instances of stigma, discrimination, and rejection were experienced by the YBLSM in this study. These accounts arose as YBLSM described why and how NPAs were helpful to them. One young man described the negative reaction of his parents when they learned of his sexual orientation:

You feel alone. When you expect your parents to always be the ones to accept anybody, no matter what…When my parents found out I was homosexual…My mom’s exact words was, “Get the hell out of my house, you demonic spirit.” Yeah, [she said] demonic spirit to me. That’s how religious they are, … I do not feel comfortable at home no more. When I get home, I’m either quiet, or I get there and get my stuff and just leave and do not show up until around 2:00 in the morning or at least 9:00 when I know they are asleep.

This young man described the way in which his parents’ initial rejection of his sexual orientation violated a fundamental expectation he held of the unconditional love parents should have for children and led to emotional distance in their relationship. Similarly, another young man described not disclosing his sexual identity to his father because his father did not express acceptance of the young man wearing nail polish and dying his hair, “My father is not-does not approve of lifestyle choices I’ve made or how I feel, so I do not tell him about any of my issues or anything going on with me, and he really does not know my sexuality. So with these people [NPAs], I feel I can trust them with things I cannot really trust in my own family.” This young man made the decision to not “come out” to his father after realizing his father did not accept forms of self-expression he considered to be outside of traditional masculine norms. That lack of emotional closeness made the young man feel closer to other adults than he did to his father.

Participants also described stigmatizing behaviors and statements made by others outside of family members that had a negative impact on BLSMY. An NPA who was an older friend of a BLSMY described the following situation that occurred at the workplace of a BLSMY involving a customer who berated him:

He works retail. He loves it. He was working in the dressing rooms and this lady did not want him back there while her daughter was changing…She called him a pedophile, actually. It was just kind of weird. He kept telling her, “Your child’s not my type.” She kept calling him a pedophile and then she did call him gay. She said that he needs to be fired. He does not need to work there…His feelings was really hurt.

Participants described rejection and discrimination BLSMY faced at home and in their communities. These events affected BLSMY emotionally, violated assumptions of parental attachment, and also, for some, resulted in housing insecurity.

### NPA Impact on Sexual Health

Similar to concepts articulated in mentoring theory, the mechanisms of NPA involvement and influence on YBLSM sexual health behavior identified in the data were their role as role models and as providers of social support, with sub-codes of esteem, emotional, informational, tangible, instrumental, and identity support (see Table 1). The range of excerpts organized into these sub-codes covered areas, such as housing, food, clothing, education, financial stability, and mental health, as well as encouraging engagement in specific sexual health behaviors, including HIV/STI testing and condom use and, for those who were HIV-positive medication adherence and attendance at medical appointments.

**Table 1:** Codes Identified in Interviews with YBLSM and Non-Parental Adults.

Parent code	Child code	Example
Stigma/Discrimination/Rejection	N/A	“A lot of people were coming in. Sorry, a family reunion, and that’s when I came out, and most people — Now usually, people I hang out with at a family reunion, that we always hang out with, did not talk to me at all, much. They said hi to me. That’s it, and when I tried to hang out with them, they made up some lame excuse to not hang out with me, like their parents told them to stay away from me and stuff.”
NPA Impact on Sexual Health NPA Impact on Contextual Influences on Sexual Health	Tangible Support	[My parents] They do not support — they say what I have is a habit and kind of a disease rather than an actual — just the same as everybody else. You just like a different gender, and my grandmother [an NPA] has taken me in. She’s cared for me and provided me with a way to succeed in life, and I really appreciate that from her.
	Instrumental Support	I also sent him information if I saw job fairs coming up, if knew of other opportunities during that time that I thought he could easily get and wasn’t terribly difficult. I also sent him scholarship and school information, just in case he was interested in going to college.
NPA Impact on Individual Influences on Sexual Health	Esteem Support	She was the mother of my first queer friend, like first really queer friend, and she would always — like she was there whenever my dad left. She knew everything about me, probably one of the closest friends/parental Figures I’ve ever known. She would look past that and help me realize like all of the things that I could do. She was like very interested — or she was like very attentive to the fact that like I’ve studied Japanese and I’ve studied music, and she would always point that out like things that I was good at and things that were interesting and would help me pursue a career later on, and I could not see that. I just would always like beat myself up over everything, but being like, I guess, I do not know, an ethnic and a sexual minority, I could not see that because I was like — I guess I had like a lot of things going on inside, and she just kind of helped me move forward, I think, with my self-esteem.
	Emotional Support	It was in the middle of a show [a school play]. One day I was downstairs, and I had like two scenes before I was supposed to go on stage, but I was downstairs, and I was just laying down on the table like basically asleep because of depression. He [an NPA who was the play director] came downstairs and was like, “What’s wrong?” I was like, “Oh, nothing. I’m just really tired.” He goes, “Are you tired because you worked so hard, or are you tired because you are sad?” I was like, “I really [do not want to] tell you.” He was like, “That’s kind of what I figured. You do realize that I am a gay man, right?” I was like, “Yes.” He goes, “Okay. You realize that I have gone through what you are going through.” “Yeah.” He goes, “You lose even more if you let those who have hurt you affect you to where you cannot perform. When it comes to you performing or you being in bed, if you let them put you in bed, you have lost.” I was like, “That’s true.” I’m a competitive person, so I was like, “I do not really like to lose.”
	Guidance	“He will date a lot of guys that are not out, well, not necessarily date but, you know, be with the guys who are not out, and you know, just how lonely that is and how upsetting and discouraging that is. And so I try to encourage him to go for what he wants, not settle, that kind of thing.”
	Identity Support	She [An NPA who was a speech coach] definitely — debate shaped me into a person that I never thought I would be. I was a lot more confident. I was a lot more articulate. I was a thinker, and we used identity, and she told me, you know, “Talk about your identity. Talk about your experience, and this is what’s going to take you far, because you have these distinct experiences that no one else has.”
	Inspiration/Role model	She [An NPA] grew up in a rural area, and it was like quite dangerous to live there, and she grew up a bit more tough and more assertive, and it’s like she’s still very — I do not want to say like feminine, but she taught me lots of things, like just because someone labels you as something or you are something, it does not mean you cannot be always something else. She says, “Just because I’m a woman, it does not mean I cannot be tough, does not mean I do not know how to fight, does not mean I cannot support my family because my husband is not working. So she was one of those things where like she’s assertive, she’s independent, she’s — it’s one of those things that makes me go, “Oh, I want to be independent. I want to be like her. I want to be able to say I can stand on my own two feet and I can be proud of that.”
NPA Impact on Sexual Health Behaviors	Guidance	In order for me to get him [an YBLSM] to take the [HIV] medicine at this point, I literally have to be, like, “Let us just take it just in case, just to make sure that it’s okay. Do not you want to be rather safe than sorry?” He’d be, like, ‘Yeah.”

### NPA Impact on Contextual and Individual Influences on Sexual Health

NPAs were described as supporting YBLSM with a variety of structural and individual factors shown to be related to sexual health behaviors. Some NPAs were described as providing tangible support to YBLSM, including food, transportation, and in some cases shelter. For example, one NPA, a male-identified person who took on the role of gay mother, described providing tangible support in the form of housing to a YBLSM who was previously unstably housed and not attending school.

He’s missing school, the main reason was because he got put out of his parents’ home, and he did not — well, now he resides with me. Before then, he was sleeping out on park benches, meeting guys to stay at their house, and doing things to stay there and earn their trust…He would not make it to school, because he did not have a way to get there, because of the busing system… Then once he got to school, because he did not have the home backing to get the new shoes, and the new clothes, the things that the regular students would generally have…so he got picked on a lot. Then it was like, “Why should I go to school when I have these guys? People are going to give me money and stuff to do things that they want to do. Why go to school?”… and now he stays with me. We are going to school probably three times a week… we are working up towards a goal of making it every day.

This account describes the cascade of negative events that can befall young sexual minority men when forced to leave their parents’ homes, including bartering with older men for shelter and increased school bullying because of lack of economic resources, as well as the vital role an NPA can play in helping to meet their basic needs, which can, in turn, support their attendance at school.

NPAs also played salient roles in racial and sexual identity development. For example, one YBLSM discussed the ability of an NPA, a coach, to serve as a confidante because of his sexual identity. “My coach is gay too, and he’s been through the same thing I’m going [through] right now. He’s been through it, so he can relate to me. So I can kind of talk to him about anything.”

For this young man, the fact that this NPA also had a sexual minority identity provided him with a sense of commonality and made him comfortable confiding in him.

Some NPAs provided identity support that was intended to socialize a young man to the realities of functioning in academic settings in the face of societal oppression. A YBLSM recounted the following about his aunt and uncle and their efforts to help him prepare to function in settings with limited diversity, “They pointed out to me that being the only black person in most of my classes, I had to be better than everyone else.” This young man’s aunt and uncle tried to instill values related to hard work given that he may be the only person of his race in class.

The type of NPA social support most commonly discussed by participants was emotional support, particularly to mitigate psychological distress. For example, a YBLSM in high school described an NPA who helped him cope with suicidal ideation after parental rejection.

My sophomore year is when I finally decided to be like, okay, I like this guy that’s in school. …so I was like head over heels for him. …So then my parents found out. My mom wasn’t too happy about it, and I remember that the whole time, [the NPA] coached me through that… when [my parents] told me, “You cannot see him…You cannot be around him at all,” it was like it broke my heart. I had become really suicidal and very depressed…[the NPA] talked me through it, and he was like, “You have to remember that, though it may be tough right now, there are people that care about you. You cannot just leave this world. You have things to do in this world.”

For this YBLSM, an NPA was a lifeline at a time in which he was feeling despondent and isolated.

Overall, NPAs helped to provide concrete support to meet basic needs, supported young men’s racial and sexual identity development, and provided emotional support to help young men cope with emotional distress. NPAs also provided informational and instrumental support. Importantly, the areas in which NPAs had an impact have been identified by SAT as influencing sexual health behaviors. In most articulations of SAT, social support is depicted as strictly an outcome of background factors (e.g., education and employment); however, our interviews with NPAs and YBLSM suggest NPA role modeling and social support impacted contextual factors, such as education and employment. In addition, NPA social support also helped YBLSM cope with stigma, discrimination, and rejection as well as with mental health difficulties.

### NPA Impact on Sexual Health Behaviors

NPAs also were described at times as being involved in encouraging specific sexual health behaviors. One NPA described being a companion and resource to a YBLSM through the early stages of his HIV diagnosis:

Yeah, I was his support system going to those meetings [with HIV care providers]. I sat in those initial conversations with the health providers about starting meds. I’m writing it all down thinking, “Later, when we go have a hamburger or something, we are going to talk about this.” I think it was just an initial [diagnosis appointment]— it was almost like shock. I think he was in shock for a while and just could not process, but eventually he was able to and did take more responsibility for his own care…Some of the mentorship has been things like asking about care or hooking up with care coordinators and making sure he’s going to his appointments.”

This NPA was able to safeguard important medical information until the YBLSM was emotionally prepared to digest and act on it.

One NPA, a gay mother, described providing support for YBLSM along the HIV care continuum:

I go with them and they get tested. That’s the first step. Once we find out the news, we go and get the blood work done or whatever. At that point, nine times out of ten, they basically either respond one of two ways. They go into the deepest of deepest depression where I do not see them anymore. They just fall off the face of the earth. Then I have the other ones that become super clingy. They will not leave. We get their medicine. We go to the clinic…We basically take care of them…We give them proteins and we do shakes, getting weight on their body. That’s what you are going to need to live with this. You’re going to have to have muscle mass. We work out. I’m not working out, but my husband works out, so they go with him…Just to make sure they know that you can still live with it. I’m an example. I tell them I’ve got it. I live with it. If I can live with it and do everything that I want to do, it’s okay. You can live with it real good.

This NPA, and his husband, provided a range of supports related to HIV, such as accompanying YBLSM to get tested and providing nourishment, as well as acting as a role model for living healthfully with HIV.

Our interviews with NPAs and YBLSM suggest NPAs in some cases were involved in integral and direct ways with sexual health behaviors. Of note, NPAs of YBLSM who were HIV-negative were not described to a great extent in the interviews as providing support for sexual health behaviors, except for encouragement to use condoms. In addition, pre-exposure prophylaxis (PrEP) was not discussed in any of the interviews. NPAs of YBLSM living with HIV described encouraging HIV care engagement, including testing and medication adherence, key steps in the journey to viral suppression. Finally, NPAs varied in the extent to which they helped across multiple areas (e.g., support for academic performance, financial support, and identity support) as opposed to more focused forms of assistance (e.g., primarily providing emotional support).

## Discussion

The study findings indicate that in the face of multiple types of stigma, discrimination, and rejection, YBLSM in this study report the involvement of non-parental adults (NPAs) in their lives. Consistent with mentoring theory (Rhodes et al., 2006), young men and NPAs described multifaceted ways, in which NPAs were positively involved in the lives of young men through role modeling and social support. NPAs helped in a range of areas from factors that affect sexual health behaviors, such as stable housing and mental health, to providing emotional support and companionship for engagement in health behaviors.

The participant accounts of facing multiple types of stigma and rejection are consistent with studies of layered stigma and multiple minority stress among ethnic and sexual minorities (Balsam et al., 2011; Arnold et al., 2014). In addition, individuals, who occupy multiple marginalized identities, can experience intersectional stigma, such that the effect of the multiple types of discrimination they face are not only layered and cumulative but also exist at a nexus such that they cannot be understood through the experience of just one identity without reference to the others (Bowleg, 2013; Earnshaw et al., 2019). The intensified effect of occupying multiple marginalized identities lead to disrupted social relationships, decreased social capital, and compromised financial resources, which, in turn, make YBLSM vulnerable to negative sexual health outcomes (Monteiro et al., 2013; Quinn et al., 2019).

Within that context, consistent with mentoring theory (Rhodes et al., 2006), NPA involvement in the lives of YBLSM in this study took various forms and impacted a variety of areas, from learning to take care of oneself financially, to providing housing. A multifaceted, multipurpose role of NPAs has been found in studies among other youth and young adult samples (Spencer, 2007; Greeson and Bowen, 2008; Johnson and Gastic, 2015) and also was recently found in a study among young Black men who have sex with men (Reed et al., 2019). NPAs thus are in a unique position to have the potential to aid YBLSM in navigating a number of developmental and societal challenges that arise due to societal marginalization in ways that are flexible and nimble enough to address different challenges young men may be facing at any given time.

The finding that NPAs were able to influence background factors is important also because intervening on these background factors can help decrease HIV disparities. For example, formal interventions designed to promote greater economic stability have been found to be an effective HIV prevention and HIV care engagement strategy among vulnerable groups, such as sex workers and those experiencing housing insecurity (Mayo-Wilson et al., 2019; Patel et al., 2019). Similarly, scholars are beginning to report that interventions that decrease mental health distress can lead to greater engagement in HIV care and reduced viral load among PLWH (Aggarwal et al., 2019; Fabian et al., 2019).

NPAs, in some instances, were also involved directly in HIV care engagement behaviors, such as doctor appointments and medication adherence. Studies have found that social support for specific sexual health behaviors can positively impact health outcomes (Arnold et al., 2018; Hermanstyne et al., 2018). Therefore, the way in which NPAs were able to provide guidance and assistance through their organic relationships with YESM with day to day behaviors related to HIV care engagement and medication adherence speaks to the potential of NPAs to contribute to care engagement if involved in more structured interventions.

A strength of this study was its examination of a relatively novel topic, NPA support to promote sexual health among YBLSM. Beyond that, the study is unique in examining both sides of the NPA-adolescent/young adult relationship by collecting data from both YBLSM and individuals identified as NPAs. This two-sided approach facilitated an understanding of this type of relationship from the perspectives of both roles. The study included NPAs of HIV-negative and HIV-positive YBLSM which expanded the diversity of information gathered in the study.

This study also had limitations. Although both YBLSM and NPA participants reported on NPA involvement in the lives of YBLSM, in most cases, pairs of YBLSM and NPAs did not report on the same relationship, which limited the comprehensiveness of conclusions drawn about the formation and dynamics of any particular relationship. The role of NPAs in supporting YBLSM living with HIV was only reported in interviews with NPAs, which occurred after the first phase of the study in which interviews with YBLSM were completed. Although YBLSM self-reported other sexually transmitted infections and, therefore, presumably also could have discussed if they were living with HIV, since the interviewers did not directly solicit information about the HIV status of YBLSM participants or establish HIV-positive status as an inclusion criterion, the information provided about NPA support to young men who are HIV-positive is only from the perspective of NPAs. This study focused on YBLSM with NPAs or connections to a supportive adult in their school or community, and therefore, the information may not be as applicable to the most vulnerable YBLSM, those without any ties to a positive adult.

### Implications and Conclusions

Due to various ecological factors that help account for HIV disparities affecting YBLSM, interventions must take into account ways to intervene at multiple contextual levels. Findings from this research preliminarily suggest partnering with organically existing support persons that already may be influencing factors at multiple levels may lead to enhanced sustainable interventions to address both structural and individual drivers of HIV disparities. The study was conducted before information about PrEP was widely available to the general public (Holloway et al., 2017; Clement et al., 2018; Dehlin et al., 2019), particularly in the geographic region where the study was conducted, and thus, it was not discussed in interviews. However, PrEP initiation and adherence may be two additional behaviors which could be encouraged and facilitated by NPAs, which deserve additional examination.

In the future, researchers should continue examining NPA influence to help elucidate the types of NPAs that may be most helpful in particular areas, as well as what factors associated with HIV-related health behaviors may be most amenable to NPA influence. As this information becomes clearer, following a youth-initiated mentoring approach (Van Dam et al., 2021), NPAs could be recruited to participate in HIV prevention and HIV care engagement interventions, both to help intervene with social determinants of health (e.g., stable employment) and to support specific sexual health behaviors (e.g., engagement with health care providers). School and municipal policymakers may also consider offering or requiring training in LGBTQ+ and racial/ethnic diversity for school personnel and community-based organizations so that adults in those settings are prepared to provide culturally responsive support YBLSM. Continued exploration of the role of NPAs in the lives of YBLSM, from both cultural and health promotion perspectives, will allow us to identify effective ways of utilizing natural interpersonal resources to empower and improve the health of this population.

## Data Availability Statement

The data sets presented in this article are not readily available because the data are transcripts from semi-structured interviews which could be used to identify participants if read in their totality. Therefore, we will not make the data available. Requests to access the data sets should be directed to emma.sterrett@louisville.edu.

## Ethics Statement

The studies involving human participants were reviewed and approved by University of Louisville Institute Review Board. Written informed consent from the participants’ legal guardian/next of kin was not required to participate in this study in accordance with the national legislation and the institutional requirements.

## Author Contributions

ES-H contributed to conceptualization, methodology, formal analysis, investigation, writing, supervision, project administration, and funding acquisition. JD contributed to formal analysis, investigation, and writing. EC contributed to formal analysis and writing. MH contributed to investigation and formal analysis. RB contributed to writing. ER contributed to formal analysis and writing. MM contributed to formal analysis and writing. MT contributed to formal analysis. EA contributed to conceptualization, methodology, writing, and supervision. All authors contributed to the article and approved the submitted version.

## Funding

This work was supported by a National Institute on Drug Abuse supplement to promote diversity (R01DA025548) and funding through a National Institute of Mental Health training grant (5R25MH067127–12).

## Conflict of Interest

The authors declare that the research was conducted in the absence of any commercial or financial relationships that could be construed as a potential conflict of interest.

## Publisher’s Note

All claims expressed in this article are solely those of the authors and do not necessarily represent those of their affiliated organizations, or those of the publisher, the editors and the reviewers. Any product that may be evaluated in this article, or claim that may be made by its manufacturer, is not guaranteed or endorsed by the publisher.
